# Point prevalence survey of antimicrobial use in three hospitals in North-Eastern Tanzania

**DOI:** 10.1186/s13756-020-00809-3

**Published:** 2020-09-07

**Authors:** Pius G. Horumpende, Stephen E. Mshana, Elise F. Mouw, Blandina T. Mmbaga, Jaffu O. Chilongola, Quirijn de Mast

**Affiliations:** 1 Kilimanjaro Christian Medical University College, P.O. BOX 2240, Moshi, Tanzania; 2 Kilimanjaro Clinical Research Institute, Moshi, Tanzania; 3 Institute of Infectious Diseases, Military College of Medical Sciences (MCMS) and General Military Hospital (GMH), Lugalo, Dar es Salaam, Tanzania; 4 Catholic University of Health and Allied Sciences (CUHAS), Mwanza, Tanzania; 5 Department of Internal Medicine, Radboudumc Center for Infectious Diseases, Radboud University Medical Centre, Nijmegen, The Netherlands

**Keywords:** Antibiotic prescription, Antibiotic stewardship, Point prevalence survey, Antimicrobial resistance, Tanzania

## Abstract

**Background:**

Antimicrobial resistance (AMR) is one of the most urgent global health threats with low-resource countries being disproportionately affected. Targeted interventions require insight in antibiotic prescription practices. A point prevalence survey (PPS) is a well-known tool to get insight in antibiotic dispensing practices in hospitals and identify areas for improvement. Here, we describe the results of a PPS performed in a tertiary, regional and district hospital in Kilimanjaro region in Tanzania.

**Methods:**

A PPS was performed in the Kilimanjaro Christian Medical Centre (KCMC; tertiary hospital), Mawenzi (regional) and St. Joseph (district) hospital in November and December 2016. Antibiotic use in all patients admitted more than 24 h and those undergoing surgery was recorded. All clinical wards were included except the pediatrics. Data from a single ward were collected on the same day.

**Results:**

A total of 399 patients were included in the PPS: 232 patients from KCMC, 94 from Mawenzi hospital and 73 patients from St. Joseph hospital. Overall prevalence of antibiotic use was 44.0%: 38% in KCMC, 59% in Mawenzi and 63% in St. Joseph. Ceftriaxone (*n* = 94, 29.8%), metronidazole (*n* = 79, 23.9%) and other antibiotics belonging to the penicillin class (*n* = 89, 28.3%) were most commonly prescribed. Antibiotics prescribed for surgical prophylaxis were continued for more than 3 days in 57% of cases.

**Conclusion:**

Our study shows a rate of broad-spectrum antibiotic use in Tanzanian hospitals and prolonged surgical antibiotic prophylaxis being a common practice. PPS is an important tool to improve future antibiotic use in Tanzania hospitals.

## Background

Antimicrobial resistance (AMR) is a serious threat to global health and human development [1]. Low-and middle-income countries (LMICs), including those in sub-Saharan Africa, suffer the highest AMR burden [2–4]. Recent surveys in hospitals across Tanzania showed a very high prevalence of colonization or infection with multidrug resistant (MDR) bacterial pathogens [5, 6]. In 2017, the National Action Plan on antimicrobial resistance was launched in Tanzania [7]. Reduction of inappropriate antibiotic use is among its priority policy. In order to identify areas for improvement and inform future policies, better insight into current antibiotic prescribing practices in hospital inpatients is needed.

Point prevalence surveys (PPS) are a well-established tool to measure the prevalence of antibiotic use in hospitals. Results can be used to identify targets for antimicrobial stewardship teams and improve local antimicrobial prescription practices [8]. Different studies have reported PPS results in health facilities in high income countries in Europe, the United States and Australia [9–13]. In contrast, only few studies have reported PPS results from health institutes in sub-Saharan Africa. Recently, a PPS performed among hospitals throughout Botswana found a high prevalence (70.6%) of antimicrobial use, as well as different areas for improvement [14]. A PPS performed in a referral hospital in Kenya reported comparable findings [15]. Overall, these reports support urgent need for additional reports of PPS results from different levels of health care in sub-Saharan Africa. Here, we report the results of a PPS performed in a tertiary, regional and district hospital in Northeastern Tanzania.

## Methods

### Setting and study area

This study was conducted in three hospitals located in Moshi Municipality in Kilimanjaro region in North-eastern Tanzania: the Kilimanjaro Christian Medical Centre (KCMC), Mawenzi Regional Hospital and St. Joseph Council Designated Hospital. KCMC has a 640-bed capacity and is the second largest consultant referral hospital in the country, serving patients from northern and central regions of Tanzania. KCMC comprises a Medical University College, which offers undergraduate and post-graduate training. It has a well-equipped clinical laboratory for patient care with a back-up advanced biotechnology laboratory. Most patients are referrals from regional or district hospitals. Mawenzi is a government-owned hospital with a 300-bed capacity and serves mainly the community of Kilimanjaro Region. In this hospital the patients are referrals from district hospitals or health centers. St. Joseph Hospital is a Catholic Church owned and Council Designated Hospital (CDH) for Moshi Municipal Council with a 100-bed capacity. The majority of patients are attended as the first point of care.

### Study procedures

The survey took place on different days in November and December 2016. Data from inpatients admitted to the clinical wards, with the exception of the pediatric wards, were collected. Data of all patients on a single ward were collected on the same day. For all patients on a ward, it was recorded whether they used at least one systemic antibiotic at 8 a.m. on the day of the survey. In that case, additional data regarding the antibiotic use were entered. Data about antibiotic use was also recorded for patients who had undergone surgery and were administered antibiotics for surgical prophylaxis in the previous 24 h. Data were collected on a written form based on the European Centres for Disease Control and Prevention (ECDC) (https://www.ecdc.europa.eu/en/publications-data/point-prevalence-survey-healthcare-associated-infections-and-antimicrobial-use-3) standard protocol form for conducting a PPS [16]. The ECDC form was modified to make it suitable for this setting and is attached (Supplement 1). Data were retrieved from patient files and treatment charts on the wards and other information sources such as the laboratory database and the treating physician. Information collected consisted of patient characteristics, details on antibiotic use (route of administration, indication, duration of prophylaxis, diagnosis, note of prescription reason, switch from intravenous to oral), information about hospital acquired infections and whether or not laboratory investigations were done. The likelihood of an infection being present was determined by an infectious disease specialist based on the information on the forms.

### Data management and analysis

Data were doubly entered in Open-Clinica (Open-Clinica LLC, MA, US). Descriptive statistics were used to summarize findings. Analyses were done using STATA statistical software version 15 (StataCorp, College Station, TX, USA).

## Results

Data from a total of 399 patients were registered from which 232, 94 and 73 patients were from the KCMC, Mawenzi hospital and St. Joseph hospital, respectively. At the day of PPS, a total number of 176 patients (44%) were using antibiotics and 19 patients (4.8%) suffered from an active hospital acquired infection (Table 1).

**Table 1:** Demographics

Characteristic	n (%)
Patients included	399 (100)
KCMC	232 (58.1)
Mawenzi	94 (23.6)
St. Joseph	73 (18.3)
Median age in years	42
Gender	
Male	162 (40.7)
Female	233 (58.5)
Not noted	2 (0.8)
Antibiotic prescribed	176 (44)
Antibiotics on admission	223 (56)
Active Hospital acquired infection	19 (4.8)

Table 2 summarizes the prevalence of antibiotic use per hospital and per department. Overall, prevalence of antibiotic use was 38% in KCMC, 59% in Mawenzi and 63% in St. Joseph.

**Table 2:** Antibiotic use in the three hospitals

Ward	Hospital								
	KCMC			Mawenzi			St. Joseph		
	Pts on antibiotics	Total pts	Prevalence (%)	Pts on antibiotics	Total pts	Prevalence (%)	Pts on antibiotics	Total	Prevalence (%)
Medical wards	26	75	35	20	31	65	26	42	62
Surgical wards total	62	157	40	35	63	56	20	31	65
Surgery	11	34	32	19	29	66	5	8	63
Urology	9	15	60	0	1	0			
Gynecology	24	53	45	16	33	48	15	23	65
ENT	11	13	85						
Orthopedic	7	42	17						
**Total**	**88**	**232**	**38**	**55**	**94**	**59**	**46**	**73**	**63**

Table 3 lists the antibiotics most commonly prescribed. In the three hospitals combined, ceftriaxone (*n* = 94; 28.5%), metronidazole (*n* = 79, 23.9%) and penicillins (*n* = 89; 26.9%) were the most commonly prescribed. Ceftriaxone was commonly prescribed in KCMC and St. Joseph, whereas penicillins (especially ampicillin and ampicillin-cloxacillin) were more commonly prescribed in the Mawenzi regional hospital. Meropenem was used in two patients in KCMC as treatment for a skin and soft tissue infection. (Table 3).

**Table 3:** Frequencies of used antibiotics

	KCMC Number of prescriptions n (% of total)	Mawenzi Number of prescriptions n (% of total)	St. Joseph Number of prescriptions n (% of total)	Total Number of prescriptions n (% of total)
Ceftriaxone	54 (35.8)	11 (11.2)	29 (35.8)	94 (28.5)
Chloramphenicol	2 (1.4)	0 (0.0)	0 (0.0)	2 (0.6)
Ciprofloxacin	2 (1.4)	0 (0.0)	2 (2.5)	4 (1.2)
Clindamycin	0 (0.0)	1 (1.0)	0 (0.0)	1 (0.3)
Trimethoprim/sulfamethoxazole	5 (7.0)	6 (6.1)	2 (2.5)	13 (3.9)
Doxycycline	1 (0.7)	1 (1.0)	3 (3.7)	5 (1.5)
Fluconazole	6 (7.7)	2 (2.0)	2 (2.5)	10 (3.0)
Gentamicin	8 (8.4)	7 (7.1)	7 (8.6)	22 (6.6)
Macrolides	3 (2.1)	0 (0.0)	1 (1.3)	4 (1.2)
Meropenem	2 (1.4)	0 (0.0)	0 (0.0)	2 (0.6)
Metronidazole	35 (23.2)	23 (23.5)	21 (26.3)	79 (23.9)
Penicillins	32 (21.2)	43 (43.9)	14 (17.3)	89 (26.9)
Amoxicillin	11 (7.0)	5 (5.1)	2 (2.5)	18 (5.5)
Amoxicillin - flucloxacillin	1 (0.7)	0 (0.0)	0 (0.0)	1 (0.3)
Amoxicillin – clavulanic acid	3 (2.1)	0 (0.0)	0 (0.0)	3 (0.9)
Ampicillin	2 (1.4)	20 (20.4)	1 (1.2)	23 (7.0)
Ampicillin - cloxacillin	5 (3.3)	12 (12.2)	11 (13.6)	28 (8.5)
Cloxacillin	6 (4.0)	5 (5.1)	0 (0.0)	11 (3.3)
Benzylpenicillin	4 (2.6)	1 (1.0)	0 (0.0)	5 (1.5)
**Total**	**151**	**98**	**81**	**330**

The indications for antibiotics were treatment of a community acquired infection (*n* = 79; 42%), an acute hospital acquired infection (*n* = 19; 10%), surgical prophylaxis (*n* = 57; 30%) or medical prophylaxis (*n* = 1; 0.5%) and unknown reason (*n* = 21; 11%). The site of infections in community acquired infection were mostly skin and soft tissue infection (*n* = 18; 23%), lower respiratory tract (*n* = 16; 21%), genitourinary infection (*n* = 12; 15%), gastroenteritis/diarrhoea (*n* = 9; 12%) and sepsis (*n* = 4; 5%). Surgical prophylaxis was the indication for antibiotic prescription in respectively, 35, 24 and 30% in the KCMC, Mawenzi and St. Joseph. Antibiotics prescribed for surgical prophylaxis were ceftriaxone (*n* = 28; 49%), penicillins (*n* = 27; 47%), occasionally in combination with gentamicin (*n* = 1; 2%) and metronidazole (*n* = 1; 2%). Surgical prophylaxis is preferably limited to a single dose immediately before initiation of surgery [17]. However, all patients except one received multiple doses of antibiotics (Table 4) and surgical prophylaxis was continued > 3 days in more than half of patients.

**Table 4:** Surgical prophylaxis practices

	Single dose n (%)	1 day n (%)	2–3 days n (%)	> 3 days n (%)
KCMC (*N* = 30)	0 (0%)	1 (3%)	12 (40%)	17 (57%)
Mawenzi (*N* = 13)	0 (0%)	3 (23%)	2 (15%)	8 (62%)
St. Joseph (*N* = 14)	1 (7%)	1 (7%)	5 (36%)	7 (50%)
Total (*N* = 57)	1 (2%)	5 (9%)	19 (33%)	32 (56%)

Next, based on information on the survey forms, an infectious diseases specialist graded the likelihood of an infection in enrolled patients, except those receiving antibiotics for surgical prophylaxis. Overall, 36% of the antibiotics were given to cases in whom a bacterial infection was deemed unlikely (Table 5).

**Table 5:** Likelihood of infection

Infection status	KCMC (n (%))	Mawenzi (n (%))	St. Joseph (n (%))	Total (N (%))
Proven	16(25)	9(20)	4(12)	29(20)
Probable	7(11)	6(14)	11(32)	24(16)
Possible	20(31)	12(27)	10(29)	42(28)
Unlikely	22(34)	17(39)	9(26)	48(36)
Total	**65(100)**	**44(100)**	**34(100)**	**143(100)**

Additional assessments were done in KCMC: in 37 (42%) patient files, the precise reason for starting antibiotics was unclear. An antibiotic treatment plan, containing details on proposed duration of antibiotic therapy, was included in only 2% of files. Regarding additional examinations, measurement of biomarkers of inflammation (erythrocyte sedimentation rate or C-reactive protein), relevant radiology or a microbiology test were done in respectively 13 (15%), 21 (24%) and 12 (14%) of the patients. In 47% of the patients, no additional investigation had taken place. During the start of the antimicrobial treatment, only 3% (*n* = 3) of the treatment was targeted, i.e. based on the result of a bacterial culture.

## Discussion

The results of the PPS in three hospitals from different levels of healthcare in Tanzania showed a prevalence of antibiotic use in hospital inpatients of 44%. This is comparable to results from African countries in the Global PPS (50%) as well as surveys from individual sub-Saharan African countries such as Botswana 70.6% [14] and Kenya 67.7% [15].

The prevalence of antibiotic use in the tertiary hospital (KCMC) was lower than in the regional and district hospital. This might be explained by more conservative antibiotic prescription practices in a teaching hospital, but also because of a lower prevalence of patients with infectious diseases.

The third-generation cephalosporin ceftriaxone and metronidazole were the most commonly prescribed antibiotics. Ceftriaxone was prescribed for both community and hospital-acquired infections, as well as for surgical prophylaxis. The frequent use of this broad-spectrum antibiotic in KCMC and hospitals across sub-Saharan Africa was reported earlier [18, 19]. Injudicious use of ceftriaxone may contribute to the high burden of AMR, including nosocomial infections with extended spectrum beta lactamase (ESBL)-producing *Enterobacteriaceae*, and complicate empirical treatment of severe bacterial infections. In view of the high prevalence of colonization and infections with ESBL positive *Enterobacteriaceae* in Tanzanian hospitals [20–23], increasing use of carbapenems in the future can be anticipated. In an attempt to limit empiric carbapenem use, the Tanzanian National Health Insurance Fund (NHIF) implemented a policy in July 2016 that limits carbapenem coverage to infections with a positive bacterial culture and sensitivity [24]. At the time of the current PPS, carbapenem use was still infrequent.

Antibiotics for treatment of infections were frequently prescribed empirically without results of microbiologic or other examinations to guide treatment. With the increasing burden of AMR across hospitals in Tanzania, expansion of diagnostic facilities to guide antibiotic therapy in hospital inpatients is urgently needed.

Next to extensive prescription of broad-spectrum antibiotics, another identified key area for improvement was prolonged surgical prophylaxis. International guidelines recommend against prolonged surgical antibiotic prophylaxis [25]. Prolonged surgical prophylaxis is often given out of fear of surgical site infections, especially when basic hospital infection prevention and control measures are not in place [26]. Evidence from a randomized controlled, non-inferiority trial involving two rural hospitals in Tanzania, however, showed no superiority of multiple doses versus a single dose surgical prophylaxis in the prevention of surgical site infections after cesarian section [27].

Finally, documentation of an antibiotic plan was only available in the case notes of two patients. Efforts on documentation of an antibiotic plan are useful in promoting i.v. to oral orders, de-escalation strategies and avoidance of duplicate therapies [28, 29].

Our study has different limitations. First, at the time of the PPS, there was a low bed – occupancy probably because of being end of the year, giving an impression of a third of patients having been studied. Second, the PPS could not be on a single day for the entire hospital, but was performed on a single day per ward.

In conclusion, the results of the PPS show a high rate of broad-spectrum antibiotic use in Tanzanian hospitals. In addition, surgical antibiotic prophylaxis prolongation is common practice. PPS can be an important aid in improvement strategies of future antibiotic use in Tanzanian hospitals.

## Supplementary information

**Additional file 1.** Data collection tool: A modified European Centers for Disease Control and Prevention (ECDC) data collection tool for antimicrobial use.

## Data Availability

The datasets generated and/or analysed during the current study are available in Kilimanjaro Clinical Research Institute (KCRI) in Moshi, Tanzania and is available on reasonable request.

## Abbreviations

KCMC: Kilimanjaro Christian Medical Centre
KCRI: Kilimanjaro Clinical Research Institute
MCMC: Military College of Medical Sciences
GMH: General Military Hospital
CUHAS: Catholic University of Health and Allied Sciences
AMR: Antimicrobial Resistance
LMIC: Low- and Middle-Income Countries
PPS: Point Prevalence Survey
CDH: Council Designated Hospital
ECDC: European Centre for Disease Prevention and Control
CRERC: College Research and Ethics Review Committee
ESBL: Extended Spectrum Beta Lactamases
NHIF: National Health Insurance Fund

## References

[1] World Health Organization (2018). WHO Report on Surveillance of Antibiotic Consumption.

[2] World Health Organization (2018). TACKLING ANTIMICROBIAL RESISTANCE (AMR) TOGETHER Working Paper 1.0: Multisectoral coordination.

[3] Horumpende PG, Sonda TB, van Zwetselaar M, Antony ML, Tenu FF, Mwanziva CE (2018). Prescription and non-prescription antibiotic dispensing practices in part I and part II pharmacies in Moshi municipality, Kilimanjaro Region in Tanzania: A simulated clients approach. PLoS One.

[4] Storr J, Twyman A, Zingg W, Damani N, Kilpatrick C, Reilly J (2017). Core components for effective infection prevention and control programmes: new WHO evidence-based recommendations. Antimicrob Resist Infect Control.

[5] Moremi N, Claus H, Mshana SE (2016). Antimicrobial resistance pattern: a report of microbiological cultures at a tertiary hospital in Tanzania. BMC Infect Dis.

[6] Manyahi J, Matee MI, Majigo M, Moyo S, Mshana SE, Lyamuya EF (2014). Predominance of multi-drug resistant bacterial pathogens causing surgical site infections in Muhimbili national hospital, Tanzania. BMC Res Notes.

[7] Ministry of Health Tanzania (2017). The National Action Plan on Antimicrobial Resistance 2017–2022.

[8] Gharbi M, Doerholt K, Vergnano S, Bielicki JA, Paulus S, Menson E (2016). Using a simple point-prevalence survey to define appropriate antibiotic prescribing in hospitalised children across the UK. BMJ Open.

[9] Cusini A, Rampini SK, Bansal V, Ledergerber B, Kuster SP, Ruef C (2010). Different patterns of inappropriate antimicrobial use in surgical and medical units at a tertiary care hospital in Switzerland: a prevalence survey. PLoS One.

[10] Sinatra I, Carubia L, Marchese V, Aprea L, D’Alessandro N, Mammina C (2013). Prevalence survey of healthcare-associated infections and antimicrobial use at the University Hospital &quot;Paolo Giaccone&quot;, Palermo, Italy. J Prev Med Hyg.

[11] Akhloufi H, Streefkerk RH, Melles DC, de Steenwinkel JEM, Schurink CAM, Verkooijen RP (2015). Point prevalence of appropriate antimicrobial therapy in a Dutch university hospital. Eur J Clin Microbiol Infect Dis.

[12] Hecker MT, Aron DC, Patel NP, Lehmann MK, Donskey CJ (2003). Unnecessary use of antimicrobials in hospitalized patients: current patterns of misuse with an emphasis on the antianaerobic spectrum of activity. Arch Intern Med.

[13] Pulcini C, Cua E, Lieutier F, Landraud L, Dellamonica P, Roger PM (2007). Antibiotic misuse: a prospective clinical audit in a French university hospital. Eur J Clin Microbiol Infect Dis.

[14] Anand Paramadhas BD, Tiroyakgosi C, Mpinda-Joseph P, Morokotso M, Matome M, Sinkala F (2019). Point prevalence study of antimicrobial use among hospitals across Botswana; findings and implications. Expert Rev Anti-Infect Ther.

[15] Okoth C, Opanga S, Okalebo F, Oluka M, Baker Kurdi A, Godman B (2018). Point prevalence survey of antibiotic use and resistance at a referral hospital in Kenya: findings and implications. Hosp Pract (1995).

[16] European Centre for Disease Prevention and Control (2016). Point prevalence survey of healthcare-associated infections and antimicrobial use in European acute care hospitals – protocol version 5.3. European Centre for Disease Prevention and Control.

[17] Bratzler DW, Dellinger EP, Olsen KM, Perl TM, Auwaerter PG, Bolon MK (2013). Clinical practice guidelines for antimicrobial prophylaxis in surgery. Am J Heal Pharm.

[18] Sonda TB, Horumpende PG, Kumburu HH, van Zwetselaar M, Mshana SE, Alifrangis M (2019). Ceftriaxone use in a tertiary care hospital in Kilimanjaro, Tanzania: a need for a hospital antibiotic stewardship programme. PLoS One.

[19] Afriyie DK, Amponsah SK, Dogbey J, Agyekum K, Kesse S, Truter I (2017). A pilot study evaluating the prescribing of ceftriaxone in hospitals in Ghana: findings and implications. Hosp Pract.

[20] Manyahi J, Moyo SJ, Tellevik MG, Ndugulile F, Urassa W, Blomberg B (2017). Detection of CTX-M-15 beta-lactamases in Enterobacteriaceae causing hospital- and community-acquired urinary tract infections as early as 2004, in Dar Es Salaam, Tanzania. BMC Infect Dis.

[21] Marando R, Seni J, Mirambo MM, Falgenhauer L, Moremi N, Mushi MF (2018). Predictors of the extended-spectrum-beta lactamases producing Enterobacteriaceae neonatal sepsis at a tertiary hospital, Tanzania. Int J Med Microbiol.

[22] Blomberg B, Jureen R, Manji KP, Tamim BS, Mwakagile DSM, Urassa WK (2005). High rate of fatal cases of pediatric septicemia caused by gram-negative bacteria with extended-spectrum beta-lactamases in Dar Es Salaam, Tanzania. J Clin Microbiol.

[23] Sonda T, Kumburu H, van Zwetselaar M, Alifrangis M, Mmbaga BT, Lund O (2018). Prevalence and risk factors for CTX-M gram-negative bacteria in hospitalized patients at a tertiary care hospital in Kilimanjaro, Tanzania. Eur J Clin Microbiol Infect Dis.

[24] Tanzania National Health Insurance Fund (2016). Price Schedule for NHIF Accredited Health Facilities.

[25] World Health Organization. Infection prevention and control. Global guidelines on the prevention of surgical site infection: WHO; 2017.

[26] Iddi Z. PROPHYLACTIC ANTIBIOTICS PRACTICES AT MNH TxANZANIA Zuberi Iddi , MD MMed ( General Surgery ) Dissertation Muhimbili University of Health and Allied Sciences October, 2013. Dar es Salaam: Muhimbili University of Health and Allied Sciences; 2013.

[27] Westen EHMN, Kolk PR, Van Velzen CL, Unkels R, Mmuni NS, Hamisi AD (2015). Single-dose compared with multiple day antibiotic prophylaxis for cesarean section in low-resource settings, a randomized controlled, noninferiority trial. Acta Obstet Gynecol Scand.

[28] Brideau-Laughlin D, Girouard G, Levesque M, MacLaggan T, Murray J, Salmon J. A point prevalence survey of antimicrobial use: benchmarking and patterns of use to support antimicrobial stewardship efforts. Can J Hosp Pharm. 2013;66.

[29] Aldeyab MA, Kearney MP, McElnay JC, Magee FA, Conlon G, Gill D (2011). A point prevalence survey of antibiotic prescriptions: benchmarking and patterns of use. Br J Clin Pharmacol.

