# Supplementary material for: Point prevalence survey of antimicrobial use in three hospitals in North-Eastern Tanzania
Source: Antimicrob Resist Infect Control. 2020 Sep 7;9:149. doi: 10.1186/s13756-020-00809-3 (PMC7487761; doi:10.1186/s13756-020-00809-3)
Supplement: Supplementary file 1 — Additional file 1. Data collection tool: A modified European Centers for Disease Control and Prevention (ECDC) data collection tool for antimicrobial use. [file 13756_2020_809_MOESM1_ESM.docx]

|  | **Point Prevalence Survey (PPS) Questionnaire** | | | Patient Counter: ------------- |
| --- | --- | --- | --- | --- |
|  | Name of Clerk: ---------------------------------------- | | | Date:------------./------------./----------- |
| 1 | Name of Hospital: | | | KCM 1, Mawenzi 2, St. Joseph 3 |
| 2 | **Department:**   \| Medical: \| 1 \|  \| Gynecology: \| 5 \| \| --- \| --- \| --- \| --- \| --- \| \| Surgery: \| 2 \|  \| ENT: \| 6 \| \| Urology: \| 3 \|  \| Rehabilitation: \| 7 \| \| Orthopedics: \| 4 \|  \| medical ICU: \| 8 \| | | | |
| 3 | **Ward**   \| Medical: \| M1 \| M2 \|  \| Gynecology: \| G1 \| G2 \| \| --- \| --- \| --- \| --- \| --- \| --- \| --- \| \| Surgery: \| S1 \| S2 \|  \| ENT: \| ENT1 \|  \| \| Urology: \| Ur \|  \|  \| Rehabilitation: \| Rehab1 \|  \| \| Orthopedics: \| Ort \|  \|  \| medical ICU: \| Med_ICU \|  \| | | | |
| 4 | Age in Years: |  | | |
| 5 | Sex: | Male 1, Female 2 | | |
| 6 | Date of hospital Admission: -----------./------------./----------- | | | |
| 7 | Referral from: | | Home 1 Other health facility 2 | |
| 8 | If other, specify: | | Dispensary 1; Health Centre 2; District hospital 3; Regional Hospital 4 | |
| 9 | Surgery Since admission:  (Note surgery is defined as incision) | | Yes 1 (give date: ………/………/…………..)  No 2 | |
| 10 | Peripheral vascular catheter: | | Yes 1 No 2 | |
| 11 | Urinary catheter: | | Yes 1 No 2 | |
| 12 | Intubation: | | Yes 1 No 2 | |
| 13 | Antibiotics during admission: | | Yes 1 No 2 | |
| 13 | Patient receives antimicrobial(s): | | Yes 1 (go to question 14 i.e. Antibiotics table) No 2. Questionnaire stops here | |
| 14 | Antibiotics table: | | | |

| Antibiotic name | Route: | Indication | For surg prophy no. of doses | Diagnosis  (anatomical site) | 2^nd^ Diagnosis | infection | Reason antib in note | Date start antimicrobial | Changed? (+reason) | If changed: Date start 1^st^ AM | Dosage per day | | |
| --- | --- | --- | --- | --- | --- | --- | --- | --- | --- | --- | --- | --- | --- |
|  |  |  |  |  |  |  |  |  |  |  | Number of doses | Strength of one dose | Mg/g/IU |
|  |  |  |  |  |  |  |  |  |  |  |  |  |  |
|  |  |  |  |  |  |  |  |  |  |  |  |  |  |
|  |  |  |  |  |  |  |  |  |  |  |  |  |  |

**Route:** 1=IV, 2=IM, 3=oral,4=Other

**Indication**: 1= community acquired infection; 2= acute hospital acquired infection; 3=surgical prophylaxis; 4=medical prophylaxis (e.g. cotrimoxazole for HIV); 5= other; 6=unknown reason

**Surgical prophylaxis**: 1=single dose; 2=one day; 3=two or three days; 4=more than 3 days

**Diagnosis (anatomical site)**: 1=Sepsis; 2=Skin/soft tissue infection; 3=Sinuses/Middle ear/Upper respiratory tract; 4=Lower respiratory tract; 5=TBC; 6=Intra-abdominal infection/peritonitis; 7=PUD/gastritis; 8=Gastroenteritis/Diarrhea; 9=Genitourinary infection; 10=Joints (septic arthritis); 11= Muscles (pyomyositis) 12=Unknown; 13=CNS Infection; 14=Other

**Reason in notes**: 1=Y; 2=N

**Changed:** 1=no; 2= yes, escalation; 3= yes, de-escalation; 4=yes, switch IV to PO; 5=yes, adverse effects; 6=yes, unknown reason; 7=unknown

**Infection:** 1=proven; 2=probable; 3=possible; 4= unlikely

| 15 | Does Patient has active HAI: | Yes 1 (go to question 16 i.e. HAI table), No 2 |
| --- | --- | --- |
| Definition:   - infection with onset ≥ 3days after admission, or - surgical site infection criteria met (surgery in previous 30dys), or - discharged from health facility <48 hrs ago, or - < Day 3 after invasive device/procedure   AND,   - HAI criteria met on survey day - OR patient is receiving treatment for HAI and case criteria were met between D1 and survey day | | |
| 16 | **HAI table:**   \|  \| **HAI1** \| **HAI2** \| \| --- \| --- \| --- \| \| Relevant device \| 1=Yes; 2=No \| 1=Yes; 2=No \| \| If yes, which device \| CVC/PVC for BSI (1), Urinary catheter for UTI (2), Intubation for HAP (3) \| CVC/PVC for BSI (1), Urinary catheter for UTI (2), Intubation for HAP (3) \| \| Device present on admission \| 1=yes; 2=no \| 1=yes; 2=no \| \| Date of onset infection \| -----------./------------./----------- \| -----------./------------./----------- \| \| Origin infection \| Current hospital (1), Other health facility (2), Unknown (3) \| Current hospital (1), Other health facility (2), Unknown (3) \| \| HAI associated to current ward \| Yes 1, No 2, Unknown (3) \| Yes 1, No 2, Unknown (3) \| | |

| 17 | Biomarker (ESR of CRP) measured before start of antibiotic: | Yes 1, No 2 |
| --- | --- | --- |
| 18 | Treatment was……… | 1=targeted ,2= Empirical |
| 19 | Was any microbiology test done? | Yes 1 (if yes, go to question 20), No 2 |
| 20 | Which test? | Gram stain (1), Pus culture (2), Blood culture (3), Urine culture (4), Serology (5), other test (6). |
| 21 | Relevant radiology | Yes (1), No (2), Unknown (3) |
| 22 | Documentation of antibiotic plan | Yes 1, No 2 |

**End of Questionnaire**
